# Rationally Controlled Electropolymerization of Conjugated Polymers: Bridging Mechanistic Insight, In-Situ Probing, and Interface Design

**DOI:** 10.1021/acscentsci.6c00071

**Published:** 2026-02-24

**Authors:** Chiao-Ling Yu, Shyh-Chyang Luo

**Affiliations:** † Department of Materials Science and Engineering, 33561 National Taiwan University, No. 1, Sec. 4, Roosevelt Road, Taipei 10617, Taiwan

## Abstract

Electropolymerization has emerged as a versatile electrochemical strategy for creating functional films of conjugated polymers, offering molecular-level tunability and spatial control. Beyond its simplicity and reagent-free nature, this approach provides a unique window into how interfacial environments, charge-transfer kinetics, and molecular coupling collectively dictate polymer growth and functionality. Because the resulting material properties are rooted in the delicate interplay between interfacial structure and reaction mechanism, unraveling these correlations remains central to both mechanistic understanding and rational material design. Recent efforts, including copolymerization, templated electropolymerization, and layer-by-layer strategies, have expanded the accessible structural and functional landscape, yet precise control of molecular architecture at electrochemical interfaces continues to pose significant challenges. This Outlook highlights emerging mechanistic insights and underscores the transformative role of advanced *in situ* and *operando* characterization techniques in bridging nanoscale structural evolution with macroscopic electrochemical behavior, ultimately pointing toward a mechanism-guided framework for designing next-generation conjugated polymers.

## Graphical abstract

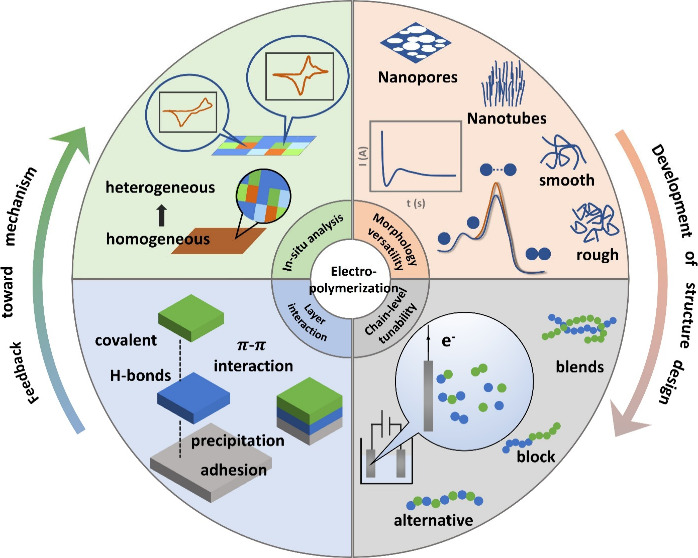

## Introduction

Electropolymerization is a versatile and controllable technique for synthesizing conjugated or functional polymer films directly on electrode surfaces.^1^ Unlike traditional chemical oxidative polymerization, which often requires strong oxidants, multistep reactions, or postprocessing steps, electropolymerization provides a more straightforward, clean, and tunable pathway for constructing polymer films.^2^ By simply adjusting electrochemical parameters such as potential, current, scan rate, and electrolyte composition, the morphology, thickness, and electrochemical behavior of the resulting conjugated polymer can be precisely regulated.^3,4^ This high degree of tunability has led to the widespread use of electropolymerization in applications such as electrochromic devices,^5^ sensors,^6^ energy storage,^7^ and electrocatalysis.^8^ Moreover, the method allows for site-selective deposition^9,10^ and compatibility with microfabrication technologies,^11^ further expanding its relevance in modern materials science and device engineering.

In this Outlook, we primarily focus on the anodic electropolymerization of heteroaromatic monomers, which proceeds through the oxidation of monomers to form radical cations, followed by coupling and deprotonation to extend the conjugated backbone.^12–14^ As the polymer grows, it becomes less soluble and more conductive, with *in situ* doping co-occurring.^15–19^ This versatile yet straightforward mechanism underpins the synthesis of polymer films from monomers such as aniline (PANI), thiophene (PTh), pyrrole (PPy), and 3,4-Ethylenedioxythiophene, EDOT (PEDOT). Electropolymerization can be performed under cyclic voltammetry (CV), constant potential, or constant current conditions, each influencing film growth differently. CV, which is often used as the first technique for elucidating the oxidation potential, allows repeated oxidation–reduction cycles,^20,21^ leading to slower but sometimes smoother deposition.^22–24^ At the same time, constant potential maintains steady radical generation and growth, allowing precise control over the amount of charge passed and, consequently, the thickness of the deposited polymer film.^25,26^ Constant current, though less common, can induce potential drift during deposition, occasionally causing overoxidation or irregular morphologies.^27^ The choice of mode therefore directly impacts nucleation, porosity, and surface uniformity of the resulting films. However, it is worth noting that the trend is not universal and can vary significantly with small changes in experimental conditions.

The mechanism of electropolymerization is best understood by jointly considering thermodynamics and kinetics, as summarized in Scheme 1. From a thermodynamic perspective, the free energy difference between the neutral monomer and its corresponding radical cation is illustrated, which is directly related to the oxidation potential and defines the minimum potential required to initiate polymerization. Once this threshold is exceeded, polymer growth becomes increasingly influenced by kinetic factors, which are commonly reflected in current–time transients during constant potential experiments. In addition, Scheme 1 qualitatively illustrates how representative experimental parameters can influence nucleation and growth behavior, ultimately leading to distinct polymer morphologies. In this outlook, we first revisit these fundamental aspects before highlighting recent advances in fabrication strategies, including copolymerization and layer-by-layer polymerization. Finally, we discuss the limitations of current methods, particularly in terms of structural control and microscale detection.

**Scheme 1:**
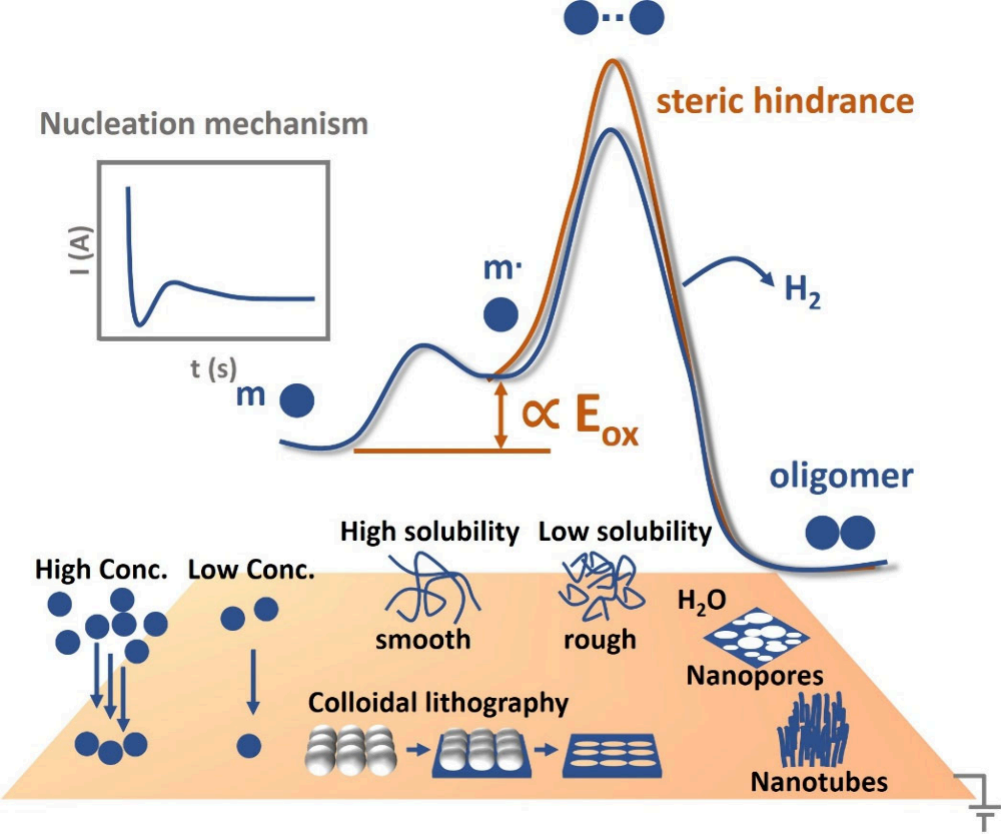
Thermodynamic and Kinetic Perspectives in Electropolymerization, Including Schematic Energy Diagram, Current-Time Transient, and Other Representative Figures of Morphology Indicated in the Paragraph

## Thermodynamic Perspective

### Oxidation Potential

The onset of polymerization requires the applied electrode potential to exceed the oxidation threshold of the monomer. In other words, applying a potential higher than the monomer’s oxidation potential ensures that the electrode Fermi level is sufficiently low to accept electrons from the monomer HOMO, providing the thermodynamic driving force for radical cation formation (Scheme 1).^28–30^ As shown in Figure 1a, structural modifications, such as introducing electron-withdrawing substituents or bulky side groups, can shift oxidation potentials and alter polymerizability.^31–33^ However, it should be noted that some monomer derivatives may fail to polymerize. For example, upon oxidation of EDOT-NH_2_, the resulting radical cation tends to localize on the amino substituent rather than on the α-position of the thiophene ring, thereby suppressing α-α coupling required for chain propagation.^34^ Similar substituent-blocking effects have also been reported in aminophenol isomers, where the substitution pattern governs accessible coupling sites and the resulting polymer structures.^35^ Beyond such structural constraints, oxidation potential itself remains a key thermodynamic parameter governing electropolymerization. In highly polar solvents, enhanced stabilization of the monomer cation could lower the oxidation potential.^36^ Reduced oxidation potentials not only facilitate polymerization but also lower the risk of overoxidation, particularly when the polymer is unstable at the potentials required for synthesis^37,38^ or susceptible to attack by hydroxyl radicals generated from water oxidation.^39^ For example, Bai et al. demonstrated that applying a potential significantly higher than the peak current potential caused polymer degradation. In contrast, potential slightly below the potential produced optimal films (Figure 1b).^23^ It should be noted that this potential depended on experimental conditions such as electrolyte composition or temperature and did not represent a universal oxidation threshold. For the second case, as there are trace amounts of water in the solvent, if the applied potential exceeds the electrochemical window of water, gas evolution (H_2_ or O_2_) may occur near the electrode. This results in hollow spheres, porous films, or even nanowires depending on the rigidity of the monomer^23,40,41^ and the acidity of the electrolyte^42,43^ (Figure 1c). For example, the resulting dense microstructures can induce superhydrophobicity with high water adhesion where water droplets remain pinned on the surface even when tilted at 90°, despite the polymer’s intrinsic hydrophilicity.^44,45^

**Figure 1:**
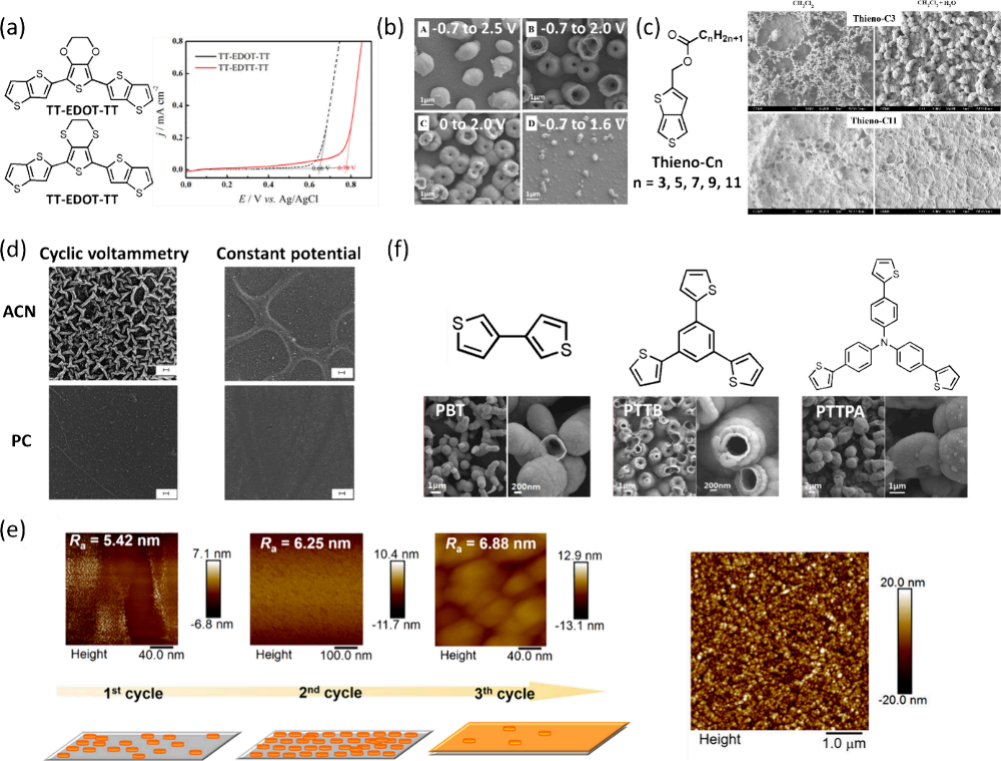
(a) Anodic polarization curves of TT-EDOT-TT and TT-EDTT-TT. Adapted with permission from ref 32. Copyright 2019, John Wiley & Sons. (b) SEM images of PTTB at different potential ranges. Reproduced from ref 23. Copyright 2018, American Chemical Society. (c) SEM images of Thieno-C3 (rigid) and Thieno-C11 (flexible) obtained in anhydrous and water-saturated DCM. Adapted with permission from ref 41. Copyright 2019, Elsevier. (d) SEM images of PEDOT using cyclic voltammetry or constant potential mode in acetonitrile or propylene carbonate. Magnification is ∼1000, scale bar is 10 μm. Adapted with permission from ref 49. Copyright 2010, American Chemical Society. (e) Schematic illustration of the Volmer–Weber growth model over three CV cycles (left) and AFM height image (5 × 5 μm^2^) showing an extremely smooth surface (right). Reproduced from ref 51. Copyright 2024, American Chemical Society. (f) SEM images of PBT, PTTB, and PTTPA. Reproduced from ref 23. Copyright 2018, American Chemical Society.

### Monomer Solubility

Upon oxidation, oligomers must precipitate onto the electrode to initiate film growth. Poorly soluble monomers or oligomers nucleate rapidly,^46^ often yielding heterogeneous films, whereas higher solubility favors the growth of longer chains and smoother morphologies. Solubility is strongly influenced by solvent polarity and temperature. Solvents with a higher dielectric constant^36^ or higher temperature^47,48^ can both enhance solubility by stabilizing charged oligomers and increasing molecular mobility, respectively. For instance, PEDOT films formed in propylene carbonate were smoother than those obtained in acetonitrile due to enhanced oligomer solubility (Figure 1d).^49^ Microscopically, the growth of longer polymer chains may follow the Volmer–Weber model. In this model, polymer nuclei form as isolated three-dimensional islands because the intermolecular interactions within polymer aggregates exceed the adhesion between oligomeric species and the substrate.^50^ These islands then grow both vertically and laterally and eventually coalesce into a continuous film. As a result, island-like microstructures can coexist within an apparently uniform film (Figure 1e).^51^ However, solubility alone does not dictate efficiency. Steric hindrance, intrinsic oxidation potential, and reactivity also strongly influence the final structure.^44,52^ Bai et al. compared three thiophene-based monomers of different sizes: BT, TTB, and TTPA. The small BT polymerized readily into long entangled nanotubes; TTB yielded well-organized nanotubes; whereas the bulky and flexible TTPA, due to steric hindrance, first formed rod-like structures that later evolved into hollow vesicles with increasing deposition scans (Figure 1f).^23^

Beyond solubility and steric effects, solvent acidity also plays a key role. Solvents with higher basicity facilitate deprotonation and accelerate polymerization.^53^ Moreover, highly polar solvents enhance reaction reversibility and doping/dedoping dynamics, improving electrochemical stability and film performance.^54^

## Kinetic Perspective

The kinetics of electropolymerization growth can be effectively monitored through the current response in constant potential conditions. Although CV provides valuable qualitative information on electropolymerization, such as oxidation potentials and electrochemical reversibility, its charging and discharging process might interfere with the continuity of the nucleation process. As a result, constant potential conditions are often preferred when quantitative kinetic analysis is required.^55,56^ From a kinetic perspective, the overall reaction rate can be limited by either charge transfer or diffusion processes. Diffusion is affected by factors such as solvent viscosity^36^ and temperature,^57^ which govern mass transport. In contrast, heterogeneous charge transfer between the electrode and monomer is intrinsically constrained by the electronic properties of the electrode material and the redox characteristics of the monomer. Thus, even under ideal diffusion conditions, the reaction rate cannot surpass this intrinsic charge transfer limit. Conversely, when the diffusion of monomers, oligomers, or counterions in the electrolyte is slower than the charge transfer rate, diffusion becomes the rate-limiting step. In practice, both processes coexist, but the dominant one determines whether the system operates under charge transfer control or diffusion control.

Electropolymerization generally proceeds in three stages (Figure 2a).^46,53,58–60^ In the initial stage, monomer oxidation generates radical cations that couple to form oligomers. Once the chains reach a critical length and become insoluble, they precipitate onto the electrode, marking the onset of nucleation and a current minimum. Stirring the solution suppresses this step, confirming the need for oligomer accumulation.^23^ In the second stage, chain propagation and precipitation accelerate, increasing the current to its maximum. In the final stage, growth and doping dominate, and the rate becomes limited by monomer diffusion from the bulk.

**Figure 2:**
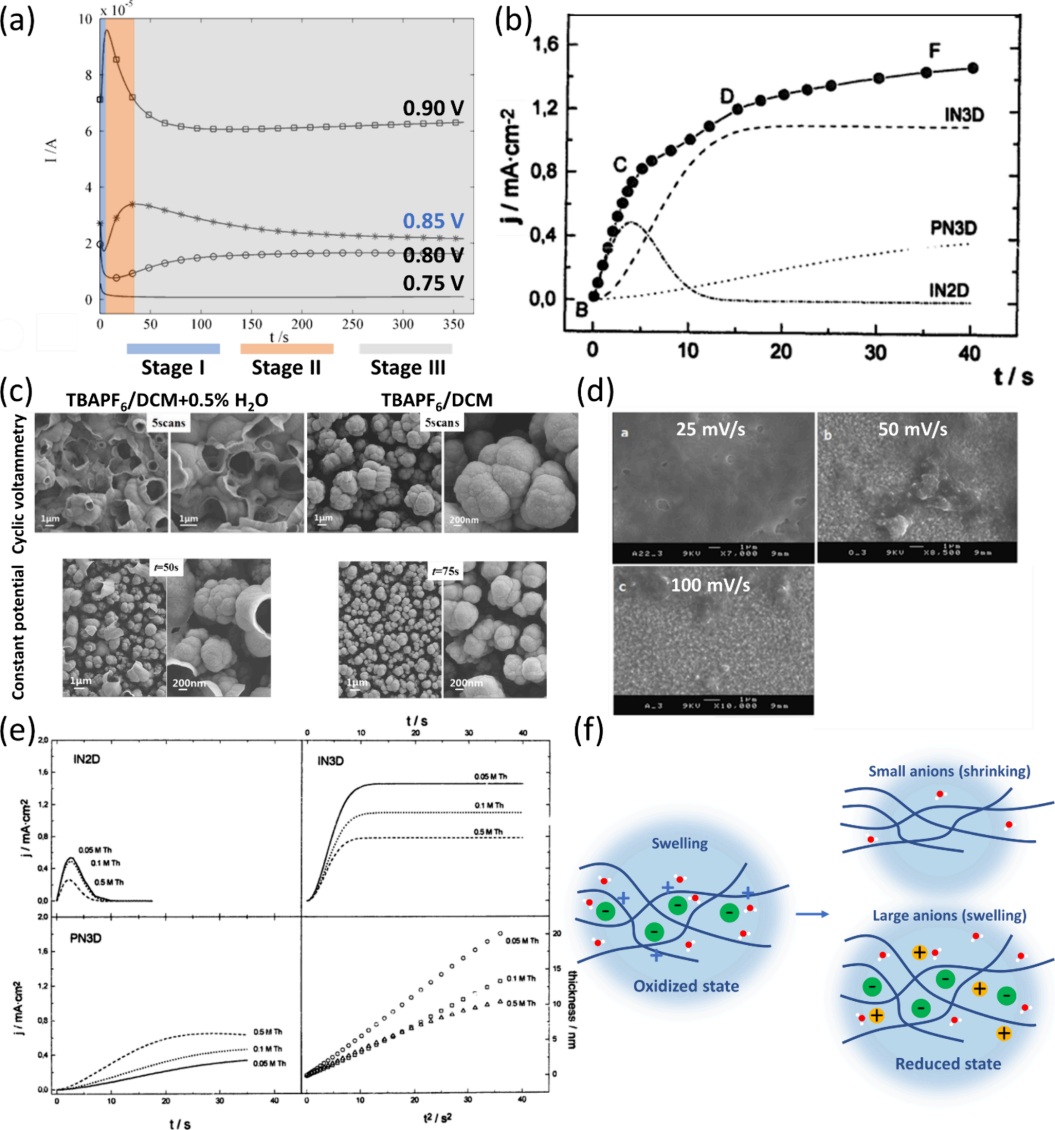
(a) Different stages of electropolymerization observed at 0.85 V. Adapted with permission from ref 58. Copyright 2004, Elsevier. (b) Current–time transient fitted by eq 1. Adapted with permission from ref 53. Copyright 1997, Elsevier. (c) SEM images of PTTB under cyclic voltammetry and constant potential modes in different electrolytes. Adapted with permission from ref 23. Copyright 2018, American Chemical Society. (d) SEM images of poly­(α-tetrathiophene) films obtained at scan rates of 25, 50, and 100 mV s^–1^. Adapted with permission from ref 67. Copyright 2013, Elsevier. (e) Effect of thiophene concentration on the isolated nucleation and growth mechanism and the thickness of the film. Adapted with permission from ref 53. Copyright 1997, Elsevier. (f) Different swelling and shrinking behavior during redox reaction (p-doping) depending on the size of anions.

The increasing current in Figure 2a could be analyzed in terms of different nucleation and growth behaviors which resemble that of metal deposition,^61,62^ as exemplified in Figure 2b. Under potentiostatic conditions, current–time transients reveal two distinct mechanisms: instantaneous nucleation (IN), where all nuclei form simultaneously and grow larger, resulting in coarser morphologies, and progressive nucleation (PN), where nuclei form continuously, yielding finer and more uniform films. Growth can proceed laterally in 2D or both laterally and vertically in 3D.^17,63^ Combining two terms together, IN2D, for example, indicates instantaneous nucleation in lateral direction. For the same monomer, the mechanism depends on experimental conditions. Therefore, quantitative analysis, which involves fitting of current–time curves, is necessary.^64^ For example, Schrebler et al. modeled the process with the following equation (Figure 2b).^53^

1 $$j=at\left[\exp\left(-{bt}^{2}\right)\right]+c\left[1-\exp\left(-{dt}^{2}\right)\right]+{et}^{-0.5}\left[1-\exp\left(-ft\right)\right]+{gt}^{-0.5}\left[1-\exp\left(-{ht}^{2}\right)\right]$$

The first two terms correspond to charge transfer-controlled IN2D and IN3D, and the latter two to diffusion-controlled IN3D (this term is included here to present the general form, following ref 64) and PN3D. The constants *a*–*h* are combinations of multiple physical quantities, including charge numbers, Faraday constant, molar mass, the density of the nucleus, the diffusion coefficient, the concentration of monomers, and the growth rate constant of the nucleus, etc. The constants represent the weighting factors and the time coefficient for each nucleation regime. By deconvoluting the current transients, the contributions of each nucleation regime can be revealed.

Their results indicated a pathway from IN2D to IN3D, and finally PN3D, as oligomers nucleate on the growing film. Figure 2b should be regarded as a representative example rather than a general electropolymerization profile.^53,65,66^

With a clear understanding of the electropolymerization kinetics, we now examine how various factors affect nucleation and film formation.

### Potential Applied

Higher potential accelerates radical cation formation, but its effect on nucleation depends on the monomer, substrate, and conditions.^68,69^ Rather than imposing a universal nucleation mechanism, the applied potential primarily modulates the relative time scales of radical formation, oligomer growth, and deposition. For example, a research study examining PTh found that higher potentials did not alter the IN2D maximum current but shifted it to an earlier time, indicating a faster transition to 3D growth due to the formation of longer oligomers.^53^ Conversely, in PPy, low potentials favored PN3D with better adhesion but poorer homogeneity, while high potentials led to IN2D.^59^

In addition to constant potential operation, parameters in CV can also influence the morphology of the resulting films. For example, the effect of the lower potential limit during cyclic voltammetry was investigated.^23^ As shown in Figure 1b, when the lower potential was varied from −0.7 to 0 V, the total amount of polymer deposited on the electrode remained nearly unchanged. However, the coverage of the top-open tube increased to almost 100%, demonstrating the ability of the voltage range to alter surface architecture. In the same literature, they also investigated the effect of water on the morphology (Figure 2c). It was further observed that under constant potential mode, the tops of the polymer tubes gradually closed after 50 s of deposition. In contrast, under CV, the porous structure remained throughout the process. Under constant potential mode, rapid polymerization resulted in dense deposition and limited gas entrapment. Under CV, slower oligomer formation allowed more time for bubble generation and stabilization. Additionally, while constant potential polymerization only produced O_2_ bubbles, CV can generate both O_2_ and H_2_, leading to more disordered and porous morphologies. The effect of scan rate has also been examined. For instance, Del Valle et al. fabricated poly­(α-tetrathiophene) at scan rates of 25, 50, and 100 mV s^–1^ (Figure 2d).^67^ Films obtained at slower rates (25 mV s^–1^) exhibited a smooth surface, whereas surface roughness increased with scan rate. The trend is commonly attributed to the shortened growth time at higher scan rates, which promote less uniform film development.^70^

### Monomer Concentration

Increasing monomer concentration generally accelerates nucleation and polymerization. Higher concentrations in PEDOT, PANI, and PPy yielded faster growth rates but rougher, less adherent films.^71,72^ Also, increasing the current slope (d*I*/d*t*) during electropolymerization could be observed.^73^ Mechanistically, higher concentrations enhance monomer diffusion to the electrode, promoting PN3D over IN2D/IN3D.^53,64^

### Electrolyte

Electrolyte anion type affects nucleation via nucleophilicity and basicity. Under potentiostatic conditions, ClO_4_^–^ and BF_4_^–^ nucleated faster than PF_6_^–^ due to stronger nucleophilicity, which promoted ion-pair formation with charged oligomers, while PF_6_^–^ enabled longer oligomer chains before precipitation.^53^ With the large amount of soluble oligomers with PF_6_^–^, monomer diffusion to the electrode surface was hindered, leading to reduced PN3D and increased contribution of IN3D, yielding thicker films. Similar CV studies showed BF_4_^–^ produced films with poor reversibility, while AsF_6_^–^ gave the fastest growth.^74^ Electrolyte concentration can also play a role in polymerization dynamics. Higher electrolyte concentrations lowered oxidation potentials and shifted control from charge transfer to diffusion due to improved conductivity and ion availability.^53,75^

Overall, changes in nucleation mechanism can also influence the thickness of the resulting films. In many reported cases, film thickness appears to correlate with the charge contribution from IN3D, as this growth mode tends to concentrate polymer mass at a limited number of nuclei rather than distributing it across a larger number of progressively formed domains.^53,65,66^ For example, in a study where thiophene electropolymerization was conducted at different monomer concentrations (Figure 2e),^53^ a higher contribution from IN3D accompanied by a reduced PN3D component, was associated with the formation of thicker films. However, because no studies to date have systematically compared films deposited under identical total charge while independently varying the relative contributions of different nucleation mechanisms, it remains premature to conclude that a higher IN3D contribution alone necessarily results in thicker films, we note that such correlations may depend on the charge associated with IN3D, rather than the contribution ratio itself.

The mechanism of electropolymerization is best understood by jointly considering thermodynamics and kinetics.

In addition to nucleation and growth kinetics, **the size and mobility** of ions critically influence the electrochemical behavior of the resulting film. When a polymer film undergoes oxidation or reduction, electrolyte ions migrate to maintain charge neutrality, resulting in the film swelling or shrinking.^76^ The size and mobility of anions significantly influence the p-doping/dedoping mechanism. In n-doping polymers, cation movement dominates the volume change. As illustrated in Figure 2f, during p-doping, the polymer backbone becomes positively charged, and anions from the electrolyte enter the film to compensate for the charge, resulting in volume expansion. In contrast, during dedoping, these anions are released back into the electrolyte, resulting in film shrinkage. However, when the electrolyte contains large or poorly mobile anions, they remain trapped, forcing cations to enter the film to balance charge, which induces swelling.^77^ In the study by Bruns et al., sodium dodecylbenzenesulfonate (NaDBS) was used as the electrolyte, where the bulky DBS^–^ anions were too immobile to participate in exchange.^78^ As a result, redox cycling proceeded mainly via Na^+^ insertion/extraction, producing a cation-driven process with smooth, reversible volume changes. For aqueous electrolytes, the hydration shell of ions must also be considered. To enter or leave the polymer matrix during doping or dedoping, ions often need to partially shed or distort their hydration shell to match the pore of the film.^79^ This dehydration or rearrangement process introduces an additional energy barrier, which becomes particularly significant for strongly hydrated ions.^80^ Consequently, hydration effects can substantially influence ion transport kinetics and swelling behavior, even when the overall doping mechanism remains the same.

These findings underscore the importance of understanding how polymerization conditions influence morphology, not only for optimizing growth but also for tailoring the functionality and performance of electroactive films in practical applications. Overall, while the examples above illustrate commonly observed trends, these behaviors are not universal. Parameters such as temperature, electrolyte composition, humidity, and substrate properties can significantly shift the optimal conditions. Therefore, the relationships summarized in this section should be viewed as tunable guidelines rather than fixed rules, requiring validation within each specific electropolymerization system.

## Advanced Fabrication Strategies

To access richer interfacial architectures, strategies such as copolymerization and layer-by-layer (LbL) polymerization have been introduced.

### Copolymerization

Copolymerization enables fine-tuning of polymer properties by combining monomers to integrate their advantages. For example, this approach is widely used in electrochromic materials to achieve a broader electrochromic color range and enhanced stability.^81–85^ For bioelectronic applications, EDOT-PC has been widely used for its zwitterion group, which suppresses nonspecific absorption. As shown in Figure 3a, cell distribution was governed by poly­(EDOT–OH-*co*-EDOT-PC) composition.^86^ Cells were preferentially confined to poly­(EDOT–OH) domains. Moreover, by copolymerization with EDOT-HQ, cell attachment became redox-controllable (Figure 3b).^87^ In electrocatalysis, a PPy-PANI (1:10) composite balanced PANI’s high surface area with PPy’s dense structure with high conductivity (Figure 3c), lowering the Tafel slope for the hydrogen evolution reaction (HER) to 144 mV/dec versus 314 and 340 mV/dec for pure PANI and PPy.^88^ Copolymers can also tailor ion affinity and morphology. Incorporating oligoethylene glycol-functionalized EDOT (EDOT-g_4_) with EDOT increased cation binding via ether coordination.^89^ As one cation can coordinate two glycol ether units, at low EDOT-g_4_ content, cation complexation may favor interchain interactions that reinforce material stability. Whereas at high EDOT-g_4_ content, intramolecular complexation can dominate and promote suspension in water (Figure 3d). As a result, film stability, water dispersibility, surface roughness, and Young’s modulus can be controlled by composition. Similarly, varying EDOT-MeOH/EDOT ratios altered hydrogen bonding and morphology, shifting from cylinders to spheres and powders as EDOT-MeOH content decreased (Figure 3e).^90^ Adhesion could be improved by introducing dopamine into PPy, leveraging dopamine’s strong substrate interactions.^91–93^ It could also serve as a catalyst and nucleation site for PPy growth, yielding smoother, more homogeneous films (Figure 3f).^94^ Nevertheless, it remains unclear whether dopamine is covalently incorporated into the polymer backbone during electropolymerization or is merely entrapped within the PPy matrix.

**Figure 3:**
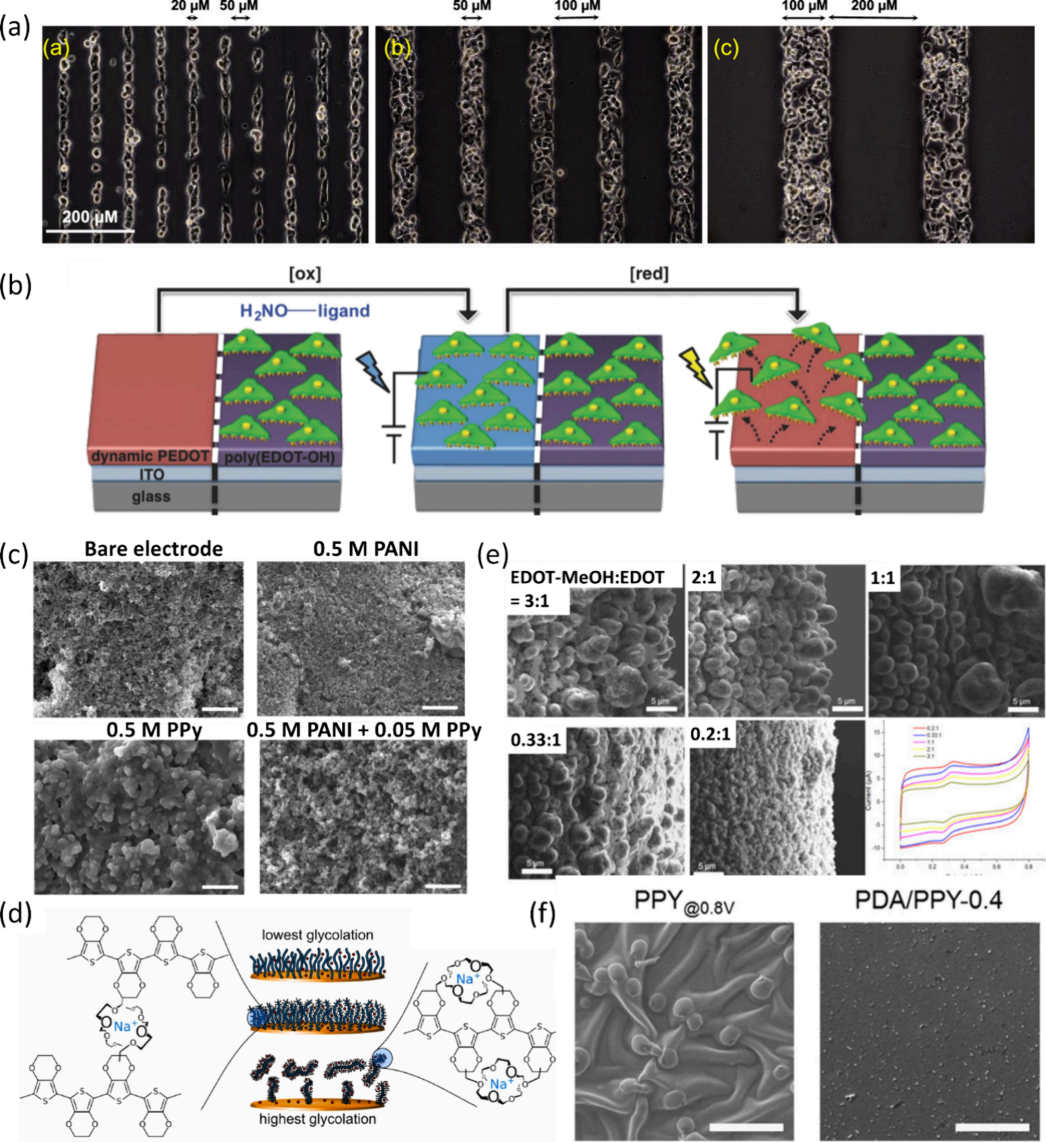
(a) Cell arrangement on substrates patterned with poly­(EDOT–OH) and poly­(EDOT–OH-*co*-EDOT-PC) stripes of varying widths. Reproduced with permission from ref 86. Copyright 2020, American Chemical Society. (b) Schematic illustration of the controlled attachment and release of cells on poly­(EDOT-HQ-*co*-EDOT-PC) film. Reproduced with permission from ref 87. Copyright 2018, John Wiley & Sons. (c) SEM images of the electrode deposited with different ratios of PANI/PPy. Adapted from ref 88. Copyright 2024 The Authors. Published by Wiley-VCH GmbH. This is an open-access article under the terms of the Creative Commons Attribution License (CC BY 4.0). (d) Correlation between degree of glycolation to cation chelation behavior. Reproduced with permission from ref 89. Copyright 2023, Elsevier. (e) SEM images and background currents of MOF525/PEM composites synthesized with various EDOT-MeOH/EDOT ratios. Adapted from ref 90. This article is distributed under the terms and conditions of the Creative Commons Attribution (CC BY) license. (f) SEM images of PPY and PDA/PPY. Reproduced from ref 94. This article is licensed under a Creative Commons Attribution 4.0 International License.

Successful simultaneous electropolymerization requires monomers with comparable polarity to be dissolved in the same solvent and to have similar oxidation potentials. Otherwise, the lower-potential monomer predominates, resulting in a homopolymer.^95^ The former issue can be solved by introducing surfactants into the electrolyte,^86^ while two strategies have been proposed to address the latter limitation. The first uses boron trifluoride ethyl ether (BFEE), a strong Lewis acid that coordinates with aromatic π-electrons, lowering oxidation potentials.^96,97^ For instance, EDOT’s potential drops from 1.0 to 1.26 to 0.5 V in BFEE.^98^ In addition to reducing the required potential, the Lewis acidic environment of BFEE may stabilize the radical cation intermediates formed during oxidation, potentially through specific solvation and/or coordination with the dopant/counterion. Such stabilization can moderate the reactivity of these intermediates and reduce uncontrolled cross-coupling typically observed in conventional solvents, thereby favoring more selective α-α coupling over competing α-β pathways and suppressing structural defects,^97,99^ although systematic experimental validation remains limited. As a result, BFEE can effectively reduce the difference in oxidation potentials between two monomers, enabling their copolymerization under a single applied potential.^52,82,83,100^ The second strategy involves lowering the oxidation potential of the high-potential monomer by increasing its degree of conjugation. Yu and Luo reduced EDOT’s potential from 0.9 to 0.5 V by forming its dimer (BiEDOT), enabling copolymerization with corannulene-(triphenylamine)_5,_ which has the oxidation potential at 0.4 V at only one-tenth of the concentration.^95^

Similar effects occur in chemical oxidative polymerization. Tran et al. found that adding a small amount of bipyrrole to pyrrole shifted the morphology from large granules to nanofibers due to bipyrrole’s faster polymerization rate.^101^ In contrast, for electropolymerization, few detailed studies have been conducted to systematically compare the electrochemical behavior of monomers and their dimers.^102^ Whether such a pronounced difference in morphology occurs in electropolymerization remains unclear and warrants further investigation.

### Layer-by-Layer Polymerization

Layer-by-layer (LbL) polymerization provides an alternative to copolymerization by integrating multiple polymers without requiring matched oxidation potentials. By electropolymerizing each monomer sequentially, LbL avoids competition during growth, enabling precise control of layer composition, thickness, and functionality.^95,103^ Compared to bulk films, LbL architectures provide more defined control over interfacial reactions, making them valuable for mechanistic electrochemical studies.^54,104^ However, LbL polymerization is quite time-consuming, and few comparative studies between copolymerization and LbL approaches have been conducted.^95,105,106^ Balser et al. constructed PPy-based bilayers with a biorepellent inner layer and a biorecognition-functionalized outer layer, achieving high selectivity even in complex samples (Figure 4a).^107^ The conductive PPy backbone further enabled sensing via conductivity changes in response to varying analyte concentrations. In this work, the precise thickness of both layers could be precisely controlled simply by the electropolymerization time. LbL polymerization could also be applied in electrochromic materials with a different purpose compared to copolymerization. For example, Firda et al. further showed that in PANI/polyacid bilayers, polyacids with lower p*K*_a_ increased doping degree and stability over a wide pH range (Figure 4b).^108^ In another work, with the inner layer serving as a color-switching film and the outer one as an ion reservoir, improved coloration efficiency and cycling stability could be achieved by self-doping from the outer layer (Figure 4c).^109^

**Figure 4:**
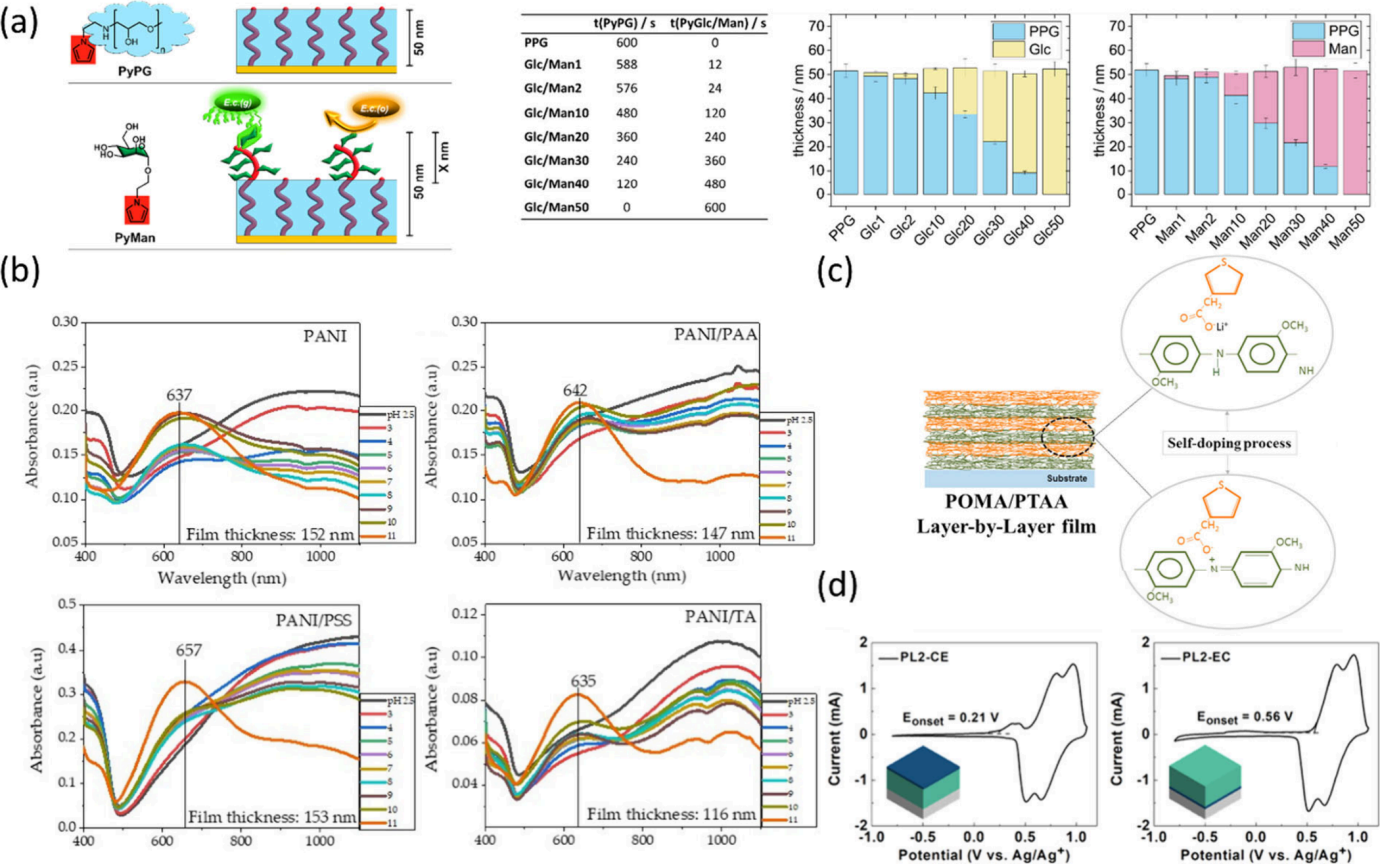
(a) Biorepellent inner layer and a biorecognition-functionalized outer layer. The thickness of each layer can be finely tuned with total thickness of 50 nm. Adapted with permission from ref 107. Copyright 2024, American Chemical Society. (b) Oxidation state transition from highly conductive ES form (absorption band around 1000 nm) to EB form (absorption peak around 640 nm depending on the dopant). ES form of PANI films was converted to EB at a pH value higher than 5, while PANI doped with acid retained the ES characteristic peak up to pH 10. Reproduced from ref 108. This article is distributed under the terms and conditions of the Creative Commons Attribution (CC BY) license. (c) Schematic representation of the self-doping effect in POMA/PTAA LbL films. Adapted with permission from ref 109. Copyright 2016, Elsevier. (d) CVs of bilayers with an outer low-potential layer and another with an inner low-potential layer, which exhibited distinct redox peaks and one peak, respectively. Adapted with permission from ref 95. Copyright 2025, American Chemical Society.

Copolymerization and LbL strategies integrate complementary monomer functionalities, moving electropolymerization beyond film growth toward the design of functional interfaces.

The order of deposition critically affects device performance. Hong et al. demonstrated that placing PANI beneath a porous PB layer enhanced redox activity due to increased ionic and electronic accessibility, demonstrating distinct properties when the depositing sequence is switched.^110^ For bilayers with distinct oxidation potential, bilayers with an outer low-potential layer exhibited distinct redox peaks from both layers during CV. In contrast, an inner low-potential layer remained silent until the outer layer was doped (Figure 4d). In conclusion, copolymerization and LbL strategies integrate complementary monomer functionalities, moving electropolymerization beyond film growth toward the design of functional interfaces. Table 1 summarizes a comparison between the two electropolymerization strategies. Key limitations of each approach are included here and discussed in greater detail in the future perspective section.

**Table 1:** Summary of the Comparison between Two Electropolymerization Strategies

Aspect	Copolymerization	LbL polymerization
Requirements	Similar oxidation potentials and solubility	Sequential redox activity and surface stability
Controllability	Feed-ratio dependent	Layer-specific and sequence defined
Risks	Homopolymer dominance, phase separation	Interlayer delamination, charge trapping
Current challenges	Molecular structure ambiguity	Interlayer interaction ambiguity
Verification methods	Averaged compositional analysis	Depth- or layer-resolved analysis
Applications	Functional materials with synergistic properties	Interfaces requiring spatial control

## Future Perspective

With the development of advanced electropolymerization techniques, promising functional materials with a broad range of monomer combinations can now be fabricated. Scheme 2a schematically illustrates this concept using a building block analogy, highlighting the expanded structural design space enabled by copolymerization and LbL polymerization. For example, Wu et al. fabricated a hydrophobic patterning on the substrate, demonstrating well-organized droplet arrays combining the photolithography technique and LbL polymerization.^111^ Meanwhile, the schematic highlights that the intrinsic structural uncertainty associated with these approaches suggests machine learning is an emerging tool to navigate this complex design space and accelerate materials discovery.

**Scheme 2:**
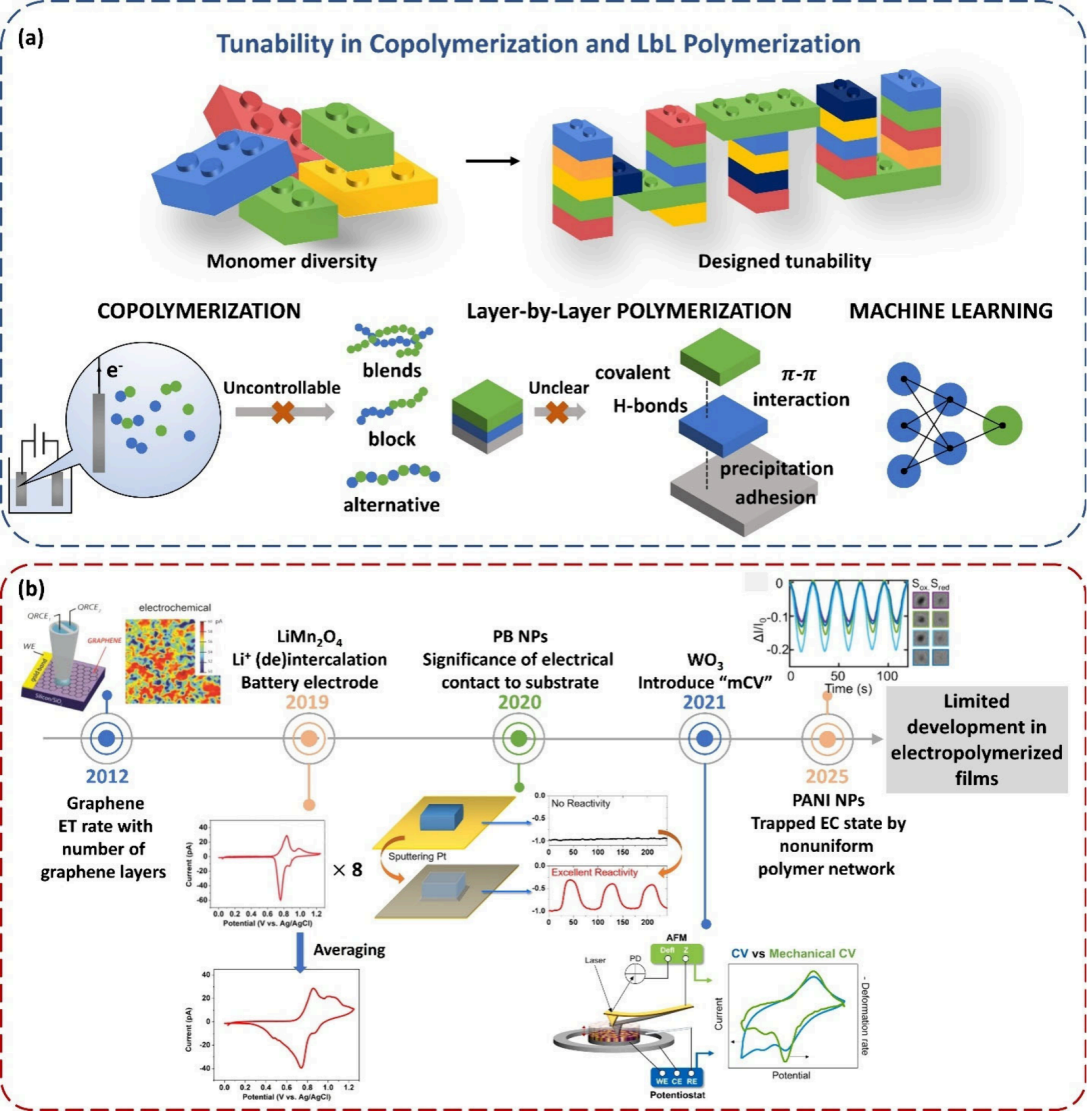
(a) Tunability and Structural Uncertainties in Copolymer and Layer-by-Layer Electropolymerization with Possible Future Directions; (b) Timeline of Studies on Local Property Detection Across Various Materials and Applications^a^

a Inorganic systems are shown below the timeline, while organic systems are displayed above. Notably, PANI nanoparticles exhibit distinct properties from electropolymerized PANI. Therefore, this study should be highlighted as a distinct case. Images adapted with permission from ref 112. Copyright 2012, American Chemical Society. Ref 113. Copyright 2019, The Authors. Published by Wiley-VCH Verlag GmbH & Co. KGaA. This is an open-access article under the terms of the Creative Commons Attribution License (CC BY 4.0). Ref 114. Copyright 2020, American Chemical Society. Ref 115. Copyright 2021, Elsevier. Ref 116. Copyright 2025, American Chemical Society.

### Ambiguity of Molecular Structure

Despite decades of development, the molecular structure of electropolymerized films remains difficult to control and characterize. Unlike chemical polymerization, where the molecular weight can be tuned by adjusting the monomer/initiator ratio, concentration, and reaction conditions, electropolymerization is constrained by the intrinsic properties of monomers. Radical stability decreases as the chain grows, limiting the degree of polymerization. Furthermore, the resulting films are typically insoluble, preventing the use of standard characterization (e.g., GPC, NMR, MALDI-TOF).^97^ While solubility can be improved through side-chain engineering, overly bulky groups may hinder polymer growth, leaving few viable options to establish reliable structure–function correlations.

Structural uncertainty is particularly evident in copolymerization and LbL polymerization, yet it is often overlooked.

Structural uncertainty is particularly evident in copolymerization and LbL polymerization, yet it is often overlooked. In practice, copolymer architecture is often inferred indirectly from CV profiles rather than being directly characterized. Although block copolymers^117,118^ and alternating copolymers^119,120^ have been reported, systematic studies on electropolymerization parameters to molecular structure remain scarce. As a result, the final structure is still largely dictated by intrinsic monomer behavior rather than tunable external parameters. The extent of phase separation and its influence on performance remain poorly understood.

Although LbL polymerization is often described as modular and controllable, the nature of interlayer interactions is not always clear. In many systems, evidence for covalent bonding between adjacent layers is lacking. The nature of the interaction between layers is a critical determinant of the overall film stability and electronic properties. For example, when nanocarbon materials are incorporated into LbL assemblies, their π-conjugated surfaces facilitate π–π electronic and hydrophobic interactions with aromatic polymers.^121,122^ For conjugated polymers without extended aromatic systems, instead of weak noncovalent interactions, covalent bonding may be required to ensure interlayer stability and efficient charge transfer.^105,123^ Various LbL fabrication techniques exist, including dip-coating, spin-coating, and self-assembly,^124,125^ where interlayer interactions are primarily based on hydrogen bonding, electrostatic attraction, or host–guest interactions.^126–128^ While covalently bonded LbL structures have been achieved through click chemistry, this requires complementary functional groups and may generate side products.^123,129,130^ In contrast, electropolymerization offers an alternative, facile, and direct route to a covalently bonded layer if suitable monomers are used, yet the extent and mechanism of such bonding remain uncertain. Indirect evidence supporting the existence of a covalent bond has existed in electrode modification for enhanced film adhesion. Because of the absence of chemical bonding between the electrode and the electropolymerized film, the resulting polymers often suffer from weak adhesion to the substrate, as evidenced by a visible color change in solution.^23^ Electrode modification with hydroxyl-functionalized EDOT^131,132^ or aryl diazonium salts^133–137^ has been shown to improve adhesion, suggesting covalent linkages between the surface and the growing polymer. However, since all LbL systems for the present are composed of derivatives of the same polymer backbone, it is unclear whether such bonding occurs when structurally different monomers are involved, or whether there is a chain length limit in the inner layer beyond which outer-layer coupling becomes ineffective.^15,16^

Resolving these uncertainties will require systematic studies that vary monomer structure, deposition sequence, and layer thickness, coupled with advanced structural and interfacial characterization. Such insights would enable more predictable, tunable architectures and a clearer understanding of structure–property relationships in electropolymerized systems. Looking ahead, integrating data-driven approaches such as machine learning may further accelerate this process by predicting material properties directly from synthesis parameters, representing an emerging but promising direction.^138^

### Local Electrochemical Activity Detection

The chemical structure of electropolymerized polymers can dramatically influence their performance.^139,140^ Even subtle modifications, such as shifting a sulfonate group along the side chain of S-EDOT, have yielded over 50-fold differences in conductivity compared to its analogue.^141^ Similarly, replacing thiophene with EDOT in an electrochromic polymer backbone produced a porous 3D network, enhancing coloration efficiency, redox kinetics, and optical contrast.^142^ Beyond molecule engineering, templates such as polystyrene beads, metal–organic frameworks (MOFs), and nanocarbons have been used to create well-defined porous structures and introduce additional functionality.^34,143–151^ For instance, in HER applications, electropolymerizing EDOT derivatives on Ni substrates with PS bead templates generated hydrophilic, porous films that exposed underlying Ni through pores smaller than gas bubbles, reducing bubble adhesion and improving gas release efficiency.^152^ Similarly, porphyrin-based MOFs grown on PEDOT films not only increase surface area but also impart molecular selectivity, enabling dopamine detection against common interferents.^90^ With the growing diversity of molecular designs and composite architectures, verifying how these structural features influence local reactivity, transport, and interfacial phenomena increasingly requires local detection techniques capable of probing chemical, morphological, and electronic heterogeneity under operating conditions.

Electropolymerized films are almost always characterized as uniform entities, despite being formed through nucleation and growth into clusters or domains of varying morphology and chemical composition.

In recent years, local imaging has predominantly focused on nanoparticles, largely due to their well-defined size, ease of detection, and modifiable surfaces.^153^ For instance, bright-field microscopy (BF) has been used to track electrochromic dynamics of individual soft PANI nanoparticles, revealing particle-to-particle variation in optical response and nonuniform nanoscale expansion during oxidation, indicating spatially heterogeneous polymer network rearrangements.^116^ Similar heterogeneity was observed for Prussian blue nanoparticles; the work highlighted the elimination of nonuniform electrical contacts to the substrate, allowing direct correlation between electrochemical activity and structural features.^114^ Tao et al. furthermore averaged the CV of 8 individual particles, resulting in similar CV resembling the CV in bulk, with individual properties being “washed out”.^113^

In contrast, electropolymerized films are almost always characterized as uniform entities, despite being formed through nucleation and growth into clusters or domains of varying morphology and chemical composition. Even advanced analytical methods such as electrochemical quartz crystal microbalance (EQCM) and *in situ* Fourier-transform infrared spectroscopy (*in situ* FTIR) often overlook this heterogeneity.^154^ Without a well-defined structure like nanoparticles, the deviations of chemical components and morphology in each micro region can still be detected. Tools such as conductive atomic force microscopy (c-AFM), scanning electrochemical microscopy (SECM), or scanning electrochemical cell microscopy (SECCM) could bridge this gap, enabling *in situ* correlation between local structure and electrochemical activity. For example, Tsai et al. highlighted the obvious difference in physical deformation detected by c-AFM over current in each pixel.^115^ In Novčić et al.’s work, a negligible relationship between the positions of hot spots for HER and the morphology of the substrate was observed, indicating the existence of other reasons.^153^ Until now, the most related research to local detection in organic systems is the correlation between electron transfer activity and the numbers of layers and stacking of graphene.^112,155^

Scheme 2b shows the timeline of studies on local property detection across various materials and applications. Early efforts and recent advances have predominantly focused on inorganic materials and nanoparticles, whereas organic and electropolymerized systems remain sparsely represented despite their growing importance in functional devices. By extending these local detection techniques from nanoparticles to electropolymerized polymers, it becomes possible to move beyond averaged bulk data and pinpoint the structure at microscale, transforming electropolymerization from a synthesis method into a platform for designing next-generation electrochemical devices.

## References

[1] Vijayakumar V., Anothumakkool B., Kurungot S., Winter M., Nair J. R. (2021). In situ
polymerization process: an essential design tool for lithium polymer
batteries. Energy Environ. Sci..

[2] Choudhary R.
B., Ansari S., Purty B. (2020). Robust electrochemical performance
of polypyrrole (PPy) and polyindole (PIn) based hybrid electrode materials
for supercapacitor application: A review. J.
Energy Storage.

[3] Chee W. K., Lim H. N., Zainal Z., Huang N. M., Harrison I., Andou Y. (2016). Flexible graphene-based
supercapacitors: a review. J. Phys. Chem. C.

[4] Lim Y. S., Tan Y., Lim H., Tan W., Mahnaz M., Talib Z., Huang N., Kassim A., Yarmo M. (2013). Polypyrrole/graphene
composite films synthesized via potentiostatic deposition. J. Appl. Polym. Sci..

[5] Tao J., Chen H., Han Y., Zhang X.-P., Peng S., Wu Z., Liu H., Liu J. (2024). Electropolymerization of DAD type
butterfly-shaped monomers based on triphenylamine-thiophene consisting
of camphor substituted quinoxaline moiety for efficient electrochromism
and supercapacitors. Eur. Polym. J..

[6] Kuntoji G., Kousar N., Gaddimath S., Koodlur Sannegowda L. (2024). Macromolecule-nanoparticle-based
hybrid materials for biosensor applications. Biosensors.

[7] Chen X., Zhang W., Zhang C., Guo Y., Yu A., Mei S., Yao C. J. (2024). Electropolymerization of Donor-Acceptor Conjugated
Polymer for Efficient Dual-Ion Storage. Adv.
Sci..

[8] Song F., Li W., Han G., Sun Y. (2018). Electropolymerization of aniline
on nickel-based electrocatalysts substantially enhances their performance
for hydrogen evolution. ACS Appl. Energy Mater..

[9] Inagi S. (2019). Site-selective
anisotropic modification of conductive objects by bipolar electropolymerization. Polym. J..

[10] Chen H., Anderson J. L., Anand R. K. (2022). Electropolymerization
of pyrrole-based
ionic liquids on selected wireless bipolar electrodes. ACS Appl. Mater. Interfaces.

[11] Synodis M., Pyo J. B., Kim M., Wang X., Allen M. G. (2021). Lithographically
patterned polypyrrole multilayer microstructures via sidewall-controlled
electropolymerization. J. Micromech. Microeng..

[12] Wei Y., Chan C. C., Tian J., Jang G. W., Hsueh K. F. (1991). Electrochemical
polymerization of thiophenes in the presence of bithiophene or terthiophene:
kinetics and mechanism of the polymerization. Chem. Mater..

[13] Mortimer R. J., Dyer A. L., Reynolds J. R. (2006). Electrochromic organic and polymeric
materials for display applications. Displays.

[14] Lange U., Roznyatovskaya N. V., Mirsky V. M. (2008). Conducting polymers in chemical sensors
and arrays. Anal. Chim. Acta.

[15] Sabouraud G., Sadki S., Brodie N. (2000). The mechanisms
of pyrrole electropolymerization. Chem. Soc.
Rev..

[16] Audebert P., Hapiot P. (1995). Fast electrochemical studies of the polymerization
mechanisms of pyrroles and thiophenes. Identification of the first
steps. Existence of π-dimers in solution. Synth. Met..

[17] Heinze J., Frontana-Uribe B. A., Ludwigs S. (2010). Electrochemistry of
Conducting Polymers—
Persistent Models and New Concepts. Chem. Rev..

[18] Sassolas A., Blum L. J., Leca-Bouvier B. D. (2012). Immobilization
strategies to develop
enzymatic biosensors. Biotechnol. Adv..

[19] Lin S., Wu Q., Lu Y. (2024). Recent progress of the application of electropolymerization
in batteries and supercapacitors: specific design of functions in
electrodes. ChemElectroChem.

[20] Rafiee M., Abrams D. J., Cardinale L., Goss Z., Romero-Arenas A., Stahl S. S. (2024). Cyclic voltammetry
and chronoamperometry: mechanistic
tools for organic electrosynthesis. Chem. Soc.
Rev..

[21] Fomo, G. ; Waryo, T. ; Feleni, U. ; Baker, P. ; Iwuoha, E. Electrochemical polymerization. In Functional Polymers; Springer, 2019; pp 105–131.

[22] Mandoj, F. ; Nardis, S. ; Di Natale, C. ; Paolesse, R. Porphyrinoid thin films for chemical sensing. In Encyclopedia of Interfacial Chemistry: Surface Science and Electrochemistry; Elsevier, 2018; pp 422–443.

[23] Bai S., Hu Q., Zeng Q., Wang M., Wang L. (2018). Variations in surface
morphologies, properties, and electrochemical responses to nitro-analyte
by controlled electropolymerization of thiophene derivatives. ACS Appl. Mater. Interfaces.

[24] Castagnola V., Bayon C., Descamps E., Bergaud C. (2014). Morphology and conductivity
of PEDOT layers produced by different electrochemical routes. Synth. Met..

[25] Mwanza C., Zhang W.-Z., Mulenga K., Ding S.-N. (2024). Advancing green
chemistry in environmental monitoring: the role of electropolymerized
molecularly imprinted polymer-based electrochemical sensors. Green Chem..

[26] Du X., Wang Z. (2003). Effects of polymerization potential on the properties of electrosynthesized
PEDOT films. Electrochim. Acta.

[27] Vogt H. (2013). On the various
types of uncontrolled potential increase in electrochemical reactors—The
anode effect. Electrochim. Acta.

[28] Elgrishi N., Rountree K. J., McCarthy B. D., Rountree E. S., Eisenhart T. T., Dempsey J. L. (2018). A practical beginner’s
guide to cyclic voltammetry. J. Chem. Educ..

[29] Cao G., Liu D. (2008). Template-based
synthesis of nanorod, nanowire, and nanotube arrays. Adv. Colloid Interface Sci..

[30] Bard, A. J. ; Faulkner, L. R. ; White, H. S. Electrochemical Methods: Fundamentals and Applications; John Wiley & Sons, 2022.

[31] Beaujuge P. M., Reynolds J. R. (2010). Color control in π-conjugated organic polymers
for use in electrochromic devices. Chem. Rev..

[32] Xue Y., Xue Z., Zhang W., Zhang W., Chen S., Lin K., Xu J. (2019). Thieno [3,
2-b] Thiophene End-Capped all-Sulfur Analog of 3, 4-Ethylenedioxythiophene
and its Eletrosynthesized Polymer: Is Distorted Conformation Not Suitable
for Electrochromism?. J. Polym. Sci., Part A:
Polym. Chem..

[33] Xu J., Zhou W., Hou J., Pu S., Yan L., Wang J. (2005). Electrosyntheses of high quality poly (5-nitroindole) films. Mater. Lett..

[34] Ouyang L., Wei B., Kuo C.-c., Pathak S., Farrell B., Martin D. C. (2017). Enhanced
PEDOT adhesion on solid substrates with electrografted P (EDOT-NH2). Sci. Adv..

[35] Barbero C., Silber J. J., Sereno L. (1989). Formation of a novel electroactive
film by electropolymerization of ortho-aminophenol: study of its chemical
structure and formation mechanism. Electropolymerization of analogous
compounds. J. Electroanal. Chem. Interfacial
Electrochem..

[36] Yadav P., Naqvi S., Patra A. (2020). Poly (3, 4-ethylenedioxyselenophene):
effect of solvent and electrolyte on electrodeposition, optoelectronic
and electrochromic properties. RSC Adv..

[37] Krische B., Zagórska M. (1989). The polythiophene
paradox. Synth.
Met..

[38] Tkach V. V., Kushnir M. V., Dytynchenko I. M., de Oliveira S. C., Luganska O. V., Ivanushko Y. G., Kovalchuk P. Y., Yagodynets P. I., Kormosh Z. O. K. Z. O. (2020). The “polythiophene
paradox”.
A theoretical sight to an alternative scenario. Appl. J. Environ. Eng. Sci..

[39] Debiemme-Chouvy C., Tran T. T. M. (2008). An insight into
the overoxidation of polypyrrole materials. Electrochem. Commun..

[40] Darmanin T., Guittard F. (2016). A one-step electrodeposition
of homogeneous and vertically
aligned nanotubes with parahydrophobic properties (high water adhesion). J. Mater. Chem. A.

[41] Sane O., Diouf A., Pan M., Moran Cruz G., Savina F., Meallet-Renault R., Dieng S. Y., Amigoni S., Guittard F., Darmanin T. (2019). Nanotubular
structures through templateless
electropolymerization using thieno [3, 4-b] thiophene derivatives
with different substituents and water content. Electrochim. Acta.

[42] Wang D., Pillier F., Cachet H., Debiemme-Chouvy C. (2022). One-pot electrosynthesis
of ultrathin overoxidized poly (3, 4-ethylenedioxythiophene) films. Electrochim. Acta.

[43] Debiemme-Chouvy C. (2009). Template-free
one-step electrochemical formation of polypyrrole nanowire array. Electrochem. Commun..

[44] Szczepanski C. R., M’Jid I., Darmanin T., Godeau G., Guittard F. (2016). A template-free
approach to nanotube-decorated polymer surfaces using 3, 4-phenylenedioxythiophene
(PhEDOT) monomers. J. Mater. Chem. A.

[45] Darmanin T., Laugier J.-P., Orange F., Guittard F. (2016). Influence of the monomer
structure and electrochemical parameters on the formation of nanotubes
with parahydrophobic properties (high water adhesion) by a templateless
electropolymerization process. J. Colloid Interface
Sci..

[46] Soto J., Díaz F., Del Valle M., Vélez J., East G. (2008). Nucleation and growth mechanisms during electropolymerization of
substituted 3-alkylthiophenes. Appl. Surf. Sci..

[47] Cysewska K., Gazda M., Jasiński P. (2017). Influence of electropolymerization
temperature on corrosion, morphological and electrical properties
of PPy doped with salicylate on iron. Surf.
Coat. Technol..

[48] Teshima K., Yamada K., Kobayashi N., Hirohashi R. (1997). Effect of
electropolymerization temperature on structural, morphological and
conductive properties of poly (aniline) deposits prepared in 1, 2-dichloroethane
without a proton donor. J. Electroanal. Chem..

[49] Poverenov E., Li M., Bitler A., Bendikov M. (2010). Major effect
of electropolymerization
solvent on morphology and electrochromic properties of PEDOT films. Chem. Mater..

[50] Ta H. Q., Perello D. J., Duong D. L., Han G. H., Gorantla S., Nguyen V. L., Bachmatiuk A., Rotkin S. V., Lee Y. H., Rümmeli M. H. (2016). Stranski-Krastanov
and Volmer-Weber CVD growth regimes
to control the stacking order in bilayer graphene. Nano Lett..

[51] Tao Y., Liu H., Kong H.-Y., Bian X.-Y., Yao B.-W., Li Y. J., Gu C., Ding X., Sun L., Han B.-H. (2024). Resistive memristors
using robust electropolymerized porous organic polymer films as switchable
materials. J. Am. Chem. Soc..

[52] Yijie T., Haifeng C., Zhaoyang Z., Xiaoqian X., Yongjiang Z. (2013). Multielectrochromic
copolymers of 3, 4-ethylenedioxythiophene and naphthalene prepared
via electropolymerization in boron trifluoride diethyl etherate. J. Electroanal. Chem..

[53] Schrebler R., Grez P., Cury P., Veas C., Merino M., Gómez H., Cordova R., Del Valle M. (1997). Nucleation
and growth mechanisms of poly (thiophene) Part 1. Effect of electrolyte
and monomer concentration in dichloromethane. J. Electroanal. Chem..

[54] Zhao M., Zhang H., Gu C., Ma Y. (2020). Electrochemical polymerization:
an emerging approach for fabricating high-quality luminescent films
and super-resolution OLEDs. J. Mater. Chem.
C.

[55] Medany S. S., Ismail K. M., Badawy W. A. (2012). Kinetics of the electropolymerization
of aminoanthraquinone from aqueous solutions and analytical applications
of the polymer film. J. Adv. Res..

[56] Yano J., Yoshikawa K.-i., Kitani A. (1997). Kinetic study of the electropolymerization
of aniline using chronoamperometric techniques. Anal. Sci..

[57] Chen Q., Wang X., Chen F., Zhang N., Ma M. (2019). Extremely
strong and tough polythiophene composite for flexible electronics. Chem. Eng. J..

[58] Pigani L., Heras A., Colina Á., Seeber R., López-Palacios J. (2004). Electropolymerisation
of 3, 4-ethylenedioxythiophene in aqueous solutions. Electrochem. Commun..

[59] Puerres J., Ortiz P., Cortés M. T. (2021). Effect
of electrosynthesis potential
on nucleation, growth, adhesion, and electronic properties of polypyrrole
thin films on fluorine-doped tin oxide (FTO). Polymers.

[60] Del
Valle M., Ugalde L., Díaz F., Bodini M., Bernède J. (2004). Effect of working conditions on the
morphology of electrosynthesized polyfuran. J. Appl. Polym. Sci..

[61] Hillman A. R., Mallen E. F. (1987). Nucleation and growtn
of polythiophene films on gold
electrodes. J. Electroanal. Chem. Interfacial
Electrochem..

[62] Downard A., Pletcher D. (1986). The influence of water on the electrodeposition
of
polypyrrole in acetonitrile. J. Electroanal.
Chem. Interfacial Electrochem..

[63] Mashreghi A., Zare H. (2016). Investigation of nucleation
and growth mechanism during electrochemical
deposition of nickel on fluorine doped tin oxide substrate. Curr. Appl. Phys..

[64] Villareal I., Morales E., Acosta J. (2001). Nucleation
and growth of LiCF3SO3-doped
polyalkylthiophenes. Polymer.

[65] Vélez J. H., Díaz F., Del Valle M., Bernède J. C., East G. (2006). Synthesis of 3′,
4′-disubstituted terthiophenes. Characterization
and electropolymerization. I. 3′, 4′-dibromo-2, 2′:
5′, 2 ″-terthiophene in photovoltaic display. J. Appl. Polym. Sci..

[66] Randriamahazaka H., Noël V., Chevrot C. (1999). Nucleation and growth of poly (3,
4-ethylenedioxythiophene) in acetonitrile on platinum under potentiostatic
conditions. J. Electroanal. Chem..

[67] Del
Valle M., Canales L., Ramos A., Díaz F. R., Hernández L., Armijo F., Bernède J., Cattin L., Louarn G. (2013). Electropolymerization and morphologic
characterization of α-tetrathiophene. Int. J. Electrochem. Sci..

[68] Castro-Beltran A., Dominguez C., Bahena-Uribe D., Sepulveda-Guzman S., Cruz-Silva R. (2014). Effect of
non-electroactive additives on the early
stage pyrrole electropolymerization on indium tin oxide electrodes. Thin Solid Films.

[69] Sayah A., Habelhames F., Bahloul A., Boudjadi A. (2021). The effect
of electrodeposition
applied potential on the electrochemical performance of polyaniline
films. J. Mater. Sci.: Mater. Electron..

[70] Babaiee M., Pakshir M., Hashemi B. (2015). Effects of potentiodynamic electropolymerization
parameters on electrochemical properties and morphology of fabricated
PANI nanofiber/graphite electrode. Synth. Met..

[71] Kulandaivalu S., Zainal Z., Sulaiman Y. (2016). Influence
of monomer concentration
on the morphologies and electrochemical properties of PEDOT, PANI,
and PPy prepared from aqueous solution. Int.
J. Polym. Sci..

[72] Conner N. R., Holubowitch N. E. (2025). Shining Light on Electropolymerization: Spectroelectrochemistry
Reveals Electrochemical-Chemical Dynamics in the Diffusion Layer and
Their Impact on Polymer Film Quality. ACS Electrochem..

[73] Atassi, Y. ; Tally, M. Electrochemical polymerization of anilinium hydrochloride. arXiv 2013, arXiv:1307.5668.

[74] Li M., Tang S., Shen F., Liu M., Li F., Lu P., Lu D., Hanif M., Ma Y. (2008). The counter anionic
size effects on electrochemical, morphological, and luminescence properties
of electrochemically deposited luminescent films. J. Electrochem. Soc..

[75] Li Y. (1997). Effect of
anion concentration on the kinetics of electrochemical polymerization
of pyrrole. J. Electroanal. Chem..

[76] Kaake L. G., Gompf B., Ludwigs S. (2023). Electrochemical and solvent-driven
swelling in a conducting polymer film. Chem.
Mater..

[77] Smela E. (2003). Conjugated
polymer actuators for biomedical applications. Adv. Mater..

[78] Bruns M., Mehraeen S., Martinez J. G., Cherif C., Jager E. W. (2025). PEDOT/Polypyrrole
Core-Sheath Fibers for Use as Conducting Polymer Artificial Muscles. ACS Appl. Mater. Interfaces.

[79] Rigo E., Dong Z., Park J. H., Kennedy E., Hokmabadi M., Almonte-Garcia L., Ding L., Aluru N., Timp G. (2019). Measurements
of the size and correlations between ions using an electrolytic point
contact. Nat. Commun..

[80] Ershov A., Xu H., Li Y., Tong T., Epsztein R. (2025). Role of Ion Dehydration
in Ion-Ion Selectivity of Dense Membranes. Environ.
Sci. Technol..

[81] Taskin A. T., Balan A., Udum Y. A., Toppare L. (2010). Improving electrochromic
properties via copolymerization. Smart Mater.
Struct..

[82] Tao Y.-j., Zhang Z.-y., Xu X.-q., Zhou Y.-j., Cheng H.-f., Zheng W.-w. (2012). Facile
and economical synthesis of high-contrast multielectrochromic
copolymers based on anthracene and 3, 4-ethylenedioxythiophene via
electrocopolymerization in boron trifluoride diethyl etherate. Electrochim. Acta.

[83] Tao Y.-j., Zhou Y.-j., Xu X.-q., Zhang Z.-y., Cheng H.-f., Zheng W.-w. (2012). A multielectrochromic
copolymer based on anthracene
and thiophene via electrochemical copolymerization in boron trifluoride
diethyl etherate. Electrochim. Acta.

[84] Ak M., Cetişli H., Toppare L. (2013). Blend or copolymer? Spectroelectrochemical
evidence of copolymerization and blending of two electrochromic monomers. Colloid Polym. Sci..

[85] Cansu
Ergun E. G., Bezgin Carbas B. (2022). Chasing the black electrochromism:
A new electrochromic copolymer based on 4, 7-bis (2, 3-dihydrothieno
[3, 4-b]­[1, 4] dioxin-5-yl) benzo [c]­[1, 2, 5] thiadiazole and ProDOT. Polymer.

[86] Zhang Y.-Q., Lin H.-A., Pan Q.-C., Qian S.-H., Zhang S.-H., Qiu G., Luo S.-C., Yu H.-h., Zhu B. (2020). Tunable protein/cell
binding and interaction with neurite outgrowth of low-impedance zwitterionic
PEDOTs. ACS Appl. Mater. Interfaces.

[87] Lin H. A., Zhu B., Wu Y. W., Sekine J., Nakao A., Luo S. C., Yamashita Y., Yu H. H. (2018). Dynamic Poly (3, 4-ethylenedioxythiophene)
s Integrate Low Impedance with Redox-Switchable Biofunction. Adv. Funct. Mater..

[88] Sharma C., Negi Y. S., Parida K., Dale S. (2024). Electro-Co-Polymerisation
of Polypyrrole-Polyaniline Composites in Ionic Liquids for Metal-Free
Hydrogen Evolution Electrodes. ChemistryOpen.

[89] Ghazal M., Susloparova A., Lefebvre C., Mansour M. D., Ghodhbane N., Melot A., Scholaert C., Guérin D., Janel S., Barois N. (2023). Electropolymerization
processing of side-chain engineered EDOT for high performance microelectrode
arrays. Biosens. Bioelectron..

[90] Chen S. S., Han P.-C., Kuok W.-K., Lu J.-Y., Gu Y., Ahamad T., Alshehri S. M., Ayalew H., Yu H.-h., Wu K. C.-W. (2020). Synthesis of
MOF525/PEDOT composites as microelectrodes
for electrochemical sensing of dopamine. Polymers.

[91] Cho G., Jang J., Moon I., Lee J.-S., Glatzhofer D. T. (1999). Enhanced
adhesion of polypyrrole film through a novel grafting method. J. Mater. Chem..

[92] Liu Y., Gan Q., Baig S., Smela E. (2007). Improving PPy adhesion by surface
roughening. J. Phys. Chem. C.

[93] Zheng W., Razal J. M., Spinks G. M., Truong V.-T., Whitten P. G., Wallace G. G. (2012). The role of unbound
oligomers in the nucleation and
growth of electrodeposited polypyrrole and method for preparing high
strength, high conductivity films. Langmuir.

[94] Kim S., Jang L. K., Park H. S., Lee J. Y. (2016). Electrochemical
deposition of conductive and adhesive polypyrrole-dopamine films. Sci. Rep..

[95] Yu C.-L., Luo S.-C. (2025). Enhanced NIR Electrochromic Properties of Corannulene-(triphenylamine)
5 and EDOT-Derived Polymers via Electrochemical Layer-by-Layer Polymerization
Compared to Copolymerization. ACS Appl. Mater.
Interfaces.

[96] Shi G., Li C., Liang Y. (1999). High-Strength Conducting Polymers Prepared by Electrochemical
Polymerization in Boron Trifluoride Diethyl Etherate Solution. Adv. Mater..

[97] Chen W., Xue G. (2005). Low potential electrochemical
syntheses of heteroaromatic conducting
polymers in a novel solvent system based on trifluroborate-ethyl ether. Prog. Polym. Sci..

[98] Cao L., Wang X.-Q., Wu Z.-X., Lu B.-Y., Shen L., Cai F.-Z., Xu J.-K., Wang J.-L., Zhang G. (2024). Fabrication
of long-term stable electrochromic device based on high-quality PEDOT
film. React. Funct. Polym..

[99] Jin S., Cong S., Xue G., Xiong H., Mansdorf B., Cheng S. Z. (2002). Anisotropic polythiophene
films with high conductivity
and good mechanical properties via a new electrochemical synthesis. Adv. Mater..

[100] Guo Y., Tao Y., Liang G., Dong M., Wang M., Hao X. (2022). Multielectrochromic
Copolymers Based on Thiophene Derivatives: Tunable
Optoelectronic Properties. Fibers Polym..

[101] Tran H. D., Shin K., Hong W. G., D’Arcy J. M., Kojima R. W., Weiller B. H., Kaner R. B. (2007). A template-free
route to polypyrrole nanofibers. Macromol. Rapid
Commun..

[102] Marrec P., Dano C., Gueguen-Simonet N., Simonet J. (1997). The anodic oxidation and polymerization of carbazoles
and dicarbazoles N-substituted by polyether chains. Synth. Met..

[103] Broncová G., Shishkanova T. V., Matějka P. (2023). The Novel
Three-Layer Electrode Based on Poly (Neutral Red) for Potentiometric
Determination of Citrates. Chemosensors.

[104] Gu M., Kim B.-S. (2021). Electrochemistry
of multilayer electrodes: from the
basics to energy applications. Acc. Chem. Res..

[105] Singhal S., Patra A. (2019). Layer-by-layer versus copolymer:
Opto-electrochemical properties of 1, 3, 5-Tris (N-carbazolyl) benzene
and EDOT based polymers. J. Electroanal. Chem..

[106] Li M., Zhang J., Nie H.-J., Liao M., Sang L., Qiao W., Wang Z. Y., Ma Y., Zhong Y.-W., Ariga K. (2013). In situ switching layer-by-layer
assembly: one-pot rapid layer assembly
via alternation of reductive and oxidative electropolymerization. Chem. Commun..

[107] Balser S., Röhrl M., Spormann C., Lindhorst T. K., Terfort A. (2024). Selective Quantification
of Bacteria in Mixtures by
Using Glycosylated Polypyrrole/Hydrogel Nanolayers. ACS Appl. Mater. Interfaces.

[108] Firda P. B. D., Malik Y. T., Oh J. K., Wujcik E. K., Jeon J.-W. (2021). Enhanced chemical and electrochemical
stability of
polyaniline-based layer-by-layer films. Polymers.

[109] Christinelli W. A., Trench A. B., Pereira E. C. (2016). Electrochromic
properties
of poly (o-methoxyaniline)-poly (3-thiophene acetic acid) layer by
layer films. Sol. Energy Mater. Sol. Cells.

[110] Hong S.-F., Hwang S.-C., Chen L.-C. (2008). Deposition-order-dependent
polyelectrochromic and redox behaviors of the polyaniline-prussian
blue bilayer. Electrochim. Acta.

[111] Wu J.-G., Lee C.-Y., Wu S.-S., Luo S.-C. (2016). Ionic liquid-assisted
electropolymerization for lithographical perfluorocarbon deposition
and hydrophobic patterning. ACS Appl. Mater.
Interfaces.

[112] Güell A. G., Ebejer N., Snowden M. E., Macpherson J. V., Unwin P. R. (2012). Structural correlations in heterogeneous electron transfer
at monolayer and multilayer graphene electrodes. J. Am. Chem. Soc..

[113] Tao B., Yule L. C., Daviddi E., Bentley C. L., Unwin P. R. (2019). Correlative
Electrochemical Microscopy of Li-Ion (De) intercalation at a Series
of Individual LiMn2O4 Particles. Angew. Chem.,
Int. Ed..

[114] Wei W., Yuan T., Jiang W., Gao J., Chen H.-Y., Wang W. (2020). Accessing the electrochemical activity of single nanoparticles by
eliminating the heterogeneous electrical contacts. J. Am. Chem. Soc..

[115] Tsai W.-Y., Wang R., Boyd S., Augustyn V., Balke N. (2021). Probing local electrochemistry via
mechanical cyclic voltammetry
curves. Nano Energy.

[116] Ma J., Wang Z., Niu B., Wang W., Wang H. (2025). Optically
Decoupling Electrochromic Dynamics and In Situ Morphological Evolution
of a Single Soft Polyaniline Nanoentity. Nano
Lett..

[117] Huang J.-J., Lin H.-A., Luo S.-C. (2023). Enhancing NIR Electrochromism
with Twisted Copolymer Films of Corannulene-Carbazole and 3, 4-Ethylenedioxythiophene. ACS Appl. Polym. Mater..

[118] Rajapakse R. G., Attanayake N. H., Karunathilaka D., Steen A. E., Hammer N. I., Strongin D. R., Watkins D. L. (2019). Advances
in electro-copolymerization of NIR emitting and electronically conducting
block copolymers. J. Mater. Chem. C.

[119] Ranathunge T. A., Karunathilaka D., Ngo D. T., Attanayake N. H., Brodgon P., Delcamp J. H., Rajapakse R. G., Watkins D. L. (2019). Radically Accessing D-A Type Ambipolar Copolymeric
Materials with Intrinsic Electrical Conductivity and Visible-Near
Infrared Absorption Via Electro-Copolymerization. Macro. Chem. Phys..

[120] Ranathunge T. A., Ngo D. T., Karunarathilaka D., Attanayake N. H., Chandrasiri I., Brogdon P., Delcamp J. H., Rajapakse R. G., Watkins D. L. (2020). Designing hierarchical structures
of complex electronically conducting organic polymers via one-step
electro-polymerization. J. Mater. Chem. C.

[121] Chakkarapani L. D., Arumugam S., Brandl M. (2021). Layer-by-layer sensor
architecture of polymers and nanoparticles for electrochemical detection
of uric acid in human urine samples. Mater.
Today Chem..

[122] Li M., Ishihara S., Akada M., Liao M., Sang L., Hill J. P., Krishnan V., Ma Y., Ariga K. (2011). Electrochemical-coupling
layer-by-layer (ECC-LbL) assembly. J. Am. Chem.
Soc..

[123] Xiao F.-X., Pagliaro M., Xu Y.-J., Liu B. (2016). Layer-by-layer
assembly of versatile nanoarchitectures with diverse dimensionality:
a new perspective for rational construction of multilayer assemblies. Chem. Soc. Rev..

[124] Izquierdo A., Ono S., Voegel J.-C., Schaaf P., Decher G. (2005). Dipping versus spraying: exploring
the deposition conditions
for speeding up layer-by-layer assembly. Langmuir.

[125] Kulandaivalu S., Sulaiman Y. (2019). Recent advances
in layer-by-layer
assembled conducting polymer based composites for supercapacitors. Energies.

[126] Kharlampieva E., Kozlovskaya V., Sukhishvili S. A. (2009). Layer-by-layer
hydrogen-bonded polymer films: from fundamentals to applications. Adv. Mater..

[127] Borges J., Mano J. F. (2014). Molecular interactions
driving the
layer-by-layer assembly of multilayers. Chem.
Rev..

[128] Ochs C. J., Such G. K., Yan Y., van Koeverden M. P., Caruso F. (2010). Biodegradable click capsules with engineered drug-loaded
multilayers. ACS Nano.

[129] Such G. K., Tjipto E., Postma A., Johnston A. P., Caruso F. (2007). Ultrathin, responsive polymer click
capsules. Nano Lett..

[130] Tian Y., He Q., Tao C., Li J. (2006). Fabrication
of fluorescent nanotubes based on layer-by-layer assembly via covalent
bond. Langmuir.

[131] Wei B., Liu J., Ouyang L., Kuo C.-C., Martin D. C. (2015). Significant
enhancement of PEDOT thin film adhesion to inorganic solid substrates
with EDOT-acid. ACS Appl. Mater. Interfaces.

[132] Huang J. J., Lin C. H., Tanaka Y., Yamamoto A., Luo S. C., Tanaka M. (2022). Manipulation of Surface Hydration
States by Tuning the Oligo (Ethylene Glycol) Moieties on PEDOT to
Achieve Platelet-Resistant Bioelectrode Applications. Adv. Mater. Interfaces.

[133] Downard A. J. (2000). Electrochemically assisted covalent modification of
carbon electrodes. Electroanalysis.

[134] Allongue P., Delamar M., Desbat B., Fagebaume O., Hitmi R., Pinson J., Savéant J.-M. (1997). Covalent
modification of carbon surfaces by aryl radicals generated from the
electrochemical reduction of diazonium salts. J. Am. Chem. Soc..

[135] Chung D.-J., Oh S.-H., Komathi S., Gopalan A. I., Lee K.-P., Choi S.-H. (2012). One-step modification of various
electrode surfaces using diazonium salt compounds and the application
of this technology to electrochemical DNA (E-DNA) sensors. Electrochim. Acta.

[136] Lo M., Diaw A. K., Gningue-Sall D., Aaron J.-J., Oturan M. A., Chehimi M. M. (2017). The role of diazonium
interface chemistry in the design
of high performance polypyrrole-coated flexible ITO sensing electrodes. Electrochem. Commun..

[137] Kumar A., Contal E., Lakard S., Dumur F., Meunier-Prest R., Viau L., Bouvet M., Lakard B. (2022). Strengthening
adhesion of polycarbazole films on ITO surface by covalent electrografting
of monomer. Surf. Interface.

[138] Yoon Y., Kim M. J., Kim J. J. (2021). Machine learning
to electrochemistry: Analysis of polymers and halide ions in a copper
electrolyte. Electrochim. Acta.

[139] Carrion S., Rodriguez-Ropero F., Aradilla D., Zanuy D., Casanovas J., Aleman C. (2010). From Poly (3, 4-ethylenedioxythiophene)
to Poly (3, 4-phenylenedioxythiophene): Impact of the Substitution
of the Ethylene Bridge by the Phenyl Ring on the Molecular Properties. J. Phys. Chem. B.

[140] Cai Y., Lu K., Murtaza I., Liu M., Sun S., Mei Z., Liu X., Xiao M., Chang S., Xu M. (2025). High-Performance electrochromic
polymers Enabled by Side-Chain engineering
for intelligent windows and supercapacitors. Eur. Polym. J..

[141] Yano H., Kudo K., Marumo K., Okuzaki H. (2019). Fully soluble
self-doped poly (3, 4-ethylenedioxythiophene) with an electrical conductivity
greater than 1000 S cm- 1. Sci. Adv..

[142] Li H., Cao J., Liu F., Zhou W., Chen X., Deng Y., Wu Z., Lu B., Mo D., Xu J., Zhang G. (2022). Stable three-dimensional
PEDOT network construction
for electrochromic-supercapacitor dual functional application. ACS Appl. Energy Mater..

[143] Briseno A. L., Han S., Rauda I. E., Zhou F., Toh C.-S., Nemanick E. J., Lewis N. S. (2004). Electrochemical
polymerization of aniline monomers infiltrated into well-ordered truncated
eggshell structures of polyelectrolyte multilayers. Langmuir.

[144] Chang N., Gu Z.-Y., Wang H.-F., Yan X.-P. (2011). Metal-organic-framework-based
tandem molecular sieves as a dual platform for selective microextraction
and high-resolution gas chromatographic separation of n-alkanes in
complex matrixes. Anal. Chem..

[145] Nellur U., Goh P. S., Padaki M., Ismail A. (2025). Recent progress
in polyMOF-based membranes and applications. J. Mater. Chem. C.

[146] Chang Y.-L., Tsai M.-D., Shen C.-H., Huang C.-W., Wang Y.-C., Kung C.-W. (2023). Cerium-based metal-organic framework-conducting
polymer nanocomposites for supercapacitors. Mater. Today Sustain..

[147] Bagheri H., Javanmardi H., Abbasi A., Banihashemi S. (2016). A metal organic
framework-polyaniline nanocomposite as a fiber coating for solid phase
microextraction. J. Chromatogr. A.

[148] Le Ouay B., Boudot M., Kitao T., Yanagida T., Kitagawa S., Uemura T. (2016). Nanostructuration of
PEDOT in porous
coordination polymers for tunable porosity and conductivity. J. Am. Chem. Soc..

[149] Xue J., Zhao F., Hu C., Zhao Y., Luo H., Dai L., Qu L. (2016). Vapor-activated
power generation on conductive polymer. Adv.
Funct. Mater..

[150] Liu J., Wang Z., Zhao Y., Cheng H., Hu C., Jiang L., Qu L. (2012). Three-dimensional graphene-polypyrrole
hybrid electrochemical actuator. Nanoscale.

[151] Chen B., Zhang Y., Lin L., Chen H., Zhao M. (2020). Au nanoparticles@
metal organic framework/polythionine loaded with
molecularly imprinted polymer sensor: Preparation, characterization,
and electrochemical detection of tyrosine. J.
Electroanal. Chem..

[152] Lin H.-H., Lin C.-H., Luo S.-C. (2023). Engineering superaerophobic
electrodes using hydrophilic PEDOT and colloidal lithography for enhanced
bubble release and efficient hydrogen evolution reaction. ACS Appl. Mater. Interfaces.

[153] Novčić K. A., Iffelsberger C., Pumera M. (2022). Layered MAX phase electrocatalyst activity is driven
by only a few hot spots. J. Mater. Chem. A.

[154] Lin C.-H., Gong Y.-C., Chen H.-Y., Wu H.-L., Luo S.-C. (2025). Real-Time
In Situ Spectroscopic and Electrochemical
Analysis of Ion-Water-Polymer Interactions at Functionalized PEDOT
Interfaces. Anal. Chem..

[155] Velicky M., Bradley D. F., Cooper A. J., Hill E. W., Kinloch I. A., Mishchenko A., Novoselov K. S., Patten H. V., Toth P. S., Valota A. T. (2014). Electron
transfer kinetics on mono-and multilayer graphene. ACS Nano.

